# Computational model for the patella onset

**DOI:** 10.1371/journal.pone.0207770

**Published:** 2018-12-11

**Authors:** Kalenia Márquez-Flórez, Sandra Shefelbine, Angélica Ramírez-Martínez, Diego Garzón-Alvarado

**Affiliations:** 1 Biomimetics Laboratory, Instituto de Biotecnología, Universidad Nacional de Colombia, Bogotá, Ciudad Universitaria, Colombia; 2 Numerical Methods and Modeling Research Group (GNUM), Universidad Nacional de Colombia, Bogotá, Ciudad Universitaria, Colombia; 3 Department of Mechanical Engineering, Northeastern University, Boston, MA, United States of America; 4 Department of Mechanical Engineering, Universidad Central, Bogotá, Colombia; University of Zaragoza, SPAIN

## Abstract

The patella is a sesamoid bone embedded within the quadriceps tendon and the patellar tendon that articulates with the femur. However, how is it formed is still unknown. Therefore, here we have evaluated, computationally, how three theories explain, independently, the patella onset. The first theory was proposed recently, in 2015. This theory suggested that the patella is initially formed as a bone eminence, attached to the anterodistal surface of the femur, while the quadriceps tendon is forming. Thereafter, a joint develops between the eminence and the femur, regulated by mechanical load. We evaluated this theory by simulating the biochemical environment that surrounds the tendon development. As a result, we obtained a patella-like structure embedded within the tendon, especially for larger flexion angles. The second and third theories are the most accepted until now. They state that the patella develops within tendons in response to the mechanical environment provided by the attaching muscles. The second theory analyzed the mechanical conditions (high hydrostatic stress) that (according to previous Carter theories) lead to the differentiation from tendon to fibrocartilage, and then, to bone. The last theory was evaluated using the self-optimizing capability of biological tissue. It was considered that the development of the patella, due to tissue topological optimization of the developing quadriceps tendon, is a feasible explanation of the patella appearance. For both theories, a patella onset was obtained as a structure embedded within the tendon. This model provided information about the relationship between the flexion angle and the patella size and shape. In conclusion, the computational models used to evaluate and analyze the selected theories allow determining that the patella onset may be the result of a combination of biochemical and mechanical factors that surround the patellar tendon development.

## Introduction

Although the development of the skeletal system has been studied over the past centuries, the development of a group of bones known as sesamoid bones is still unknown [1]. Sesamoid bones, such as the patella, are bones embedded superficially within tendons; these tendons are usually around joints. The name sesamoid comes from the Latin word *sesamum* (sesame seed), due to the small size of most of these bones, and the morphological resemblance of this bone to the seed [1]. Sesamoid bones can be found in several joints throughout the body, including hand, wrist, foot, neck, ear, and knee. The patella, or kneecap, is the largest, most recognized and studied sesamoid bone in the human body.

The patella has an important role in the stability of the knee, facilitating the function and locomotion of the lower limb [2]. It is believed that its main purpose is to increase the moment arm of the quadriceps muscle, by augmenting the distance between this muscle and the center of rotation of the knee joint [3]. The patella contributes to the compressive force distribution of the patella-femoral joint by increasing the contact area during flexion of the knee [3]. In addition, the patella protects the quadriceps tendon from high stress [4], while centralizing the pull of the quadriceps muscle complex, protecting the knee from dislocating [3].

The accepted theory on the development of the patella is that it develops inside the tendon in response to mechanical stimuli evoked when the muscles contract [1,5,6]. This theory is based on the idea that mechanical stimuli play a crucial role in tissue differentiation [7]. Therefore, while the tendon is immature, a zone of the tendon is subjected to high hydrostatic stress and low tensile strain. This leads to the differentiation of the fibrous tissue into cartilage and then to the ossification of the cartilage to form the patella.

According to the aforementioned tissue adaptation theory, topological optimization may also be suitable to explain the development of the patella. Topology optimization distributes the material in a design domain through the minimization of the strain energy; as a result, this material redistribution produces an optimal configuration for low energy consumption [8]. This theory has been widely used in many engineering and biology fields, such as the architecture of the proximal femur [9–12] as well as in the design of scaffolds, implants, bone replacements, and prostheses [13–16].

On the other hand, a recent study [1] proposed that the patella initially develops as part of the femur, similar to the way in which a bone eminence is developed. This eminence is initially formed by progenitor cells that express both Sox9 and Scx. This mechanism is controlled by Transforming Growth Factor-Beta (TGF-β) and Bone Morphogenetic Protein-4 (BMP-4), which determine the differentiation of chondrogenic cells. According to this theory, the eminence is separated from the preexisting cartilage femur under different environmental conditions, but they consider more plausible that the patella may be separated from the femur because of the presence of a remaining joint inducer molecule on the epiphysis. As a result of this process, a new bone embedded within the tendon is formed, thus creating a sesamoid bone, which will be the patella [1].

Currently, to the best of the authors’ knowledge, there is not an accepted (or unified) mechanism in the literature that explains the formation of sesamoid bones. In fact, the development of the patella could result from the combination of the above-mentioned theories. The aim of this work is to evaluate, separately, the outcome of three conceptual computational models for the development of the kneecap. The first model considers the biochemical aspects present on the onset of the quadriceps tendon and patella. The second model examines the cell behavior under the mechanical stimuli present during the formation of this sesamoid bone. The last model optimizes the mechanical environment of the tissues based on minimizing strain energy (topological optimization).

The computational models allow a comparison of the three theories of patellar development, demonstrating the strengths and the plausibility of each theory. These models provide a stepping stone to establish a unified theory about the onset of this bone, which might be able to predict patellar-associated diseases.

## Theory I: Biochemical theory

**Fig 1:**
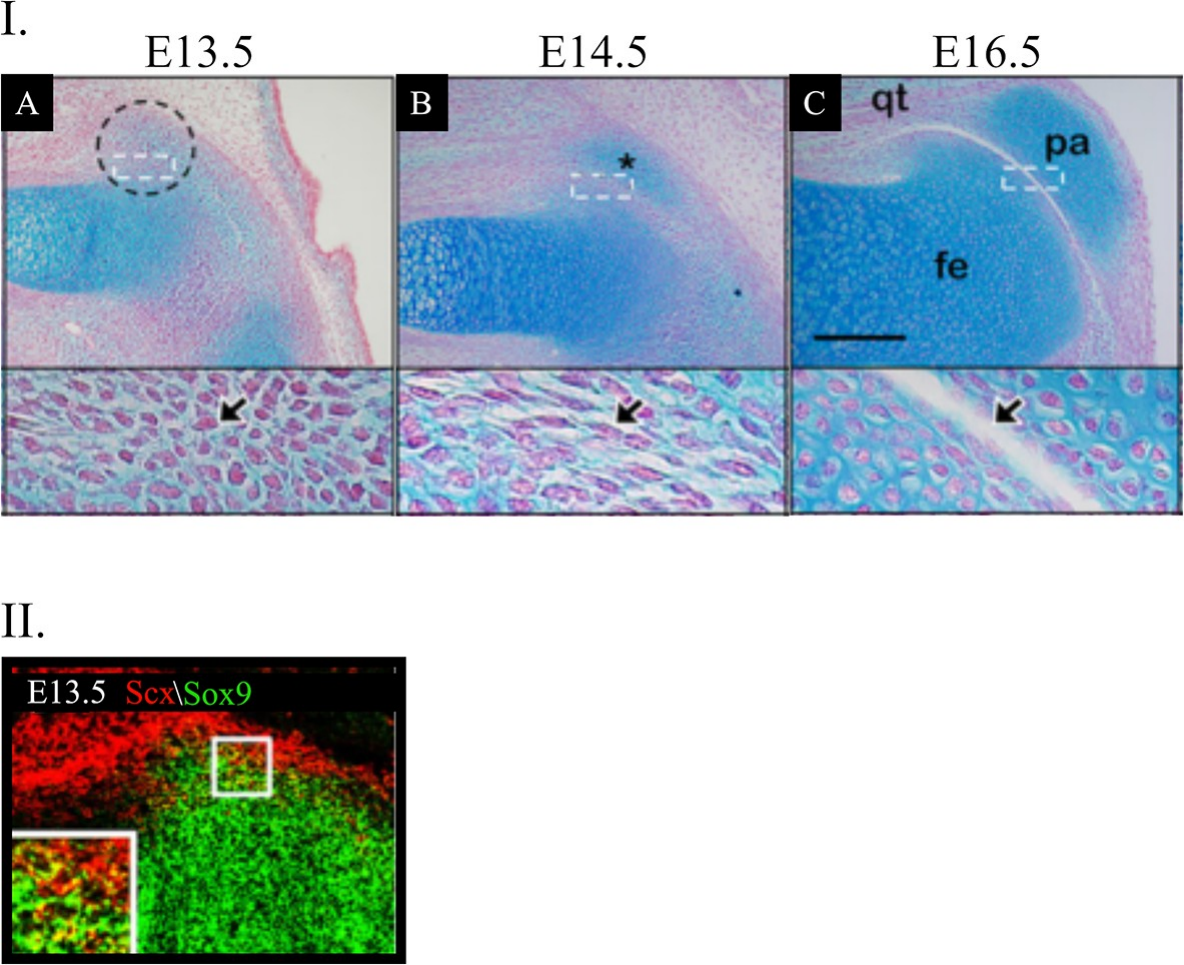
Sagittal sections of the patella from hindlimbs of wild-type embryos stained with Alcian Blue and Fast Red to highlight cartilage cells (I. A-C). (I. A) At E13.5, an aggregation of chondrogenic cells is seen at the presumable location of the patella (dashed circles) that appears to be part of the femur, as the boundary cells are absent (arrows). (I. B) At E14.5, although the patella (*) and femur are distinguishable, the patellofemoral joint is missing. The boundary between the two cartilaginous elements is occupied by cells with distinct flat and elongated morphology (arrow). At E16.5 (I. A), the patella (pa) appears as a distinct cartilaginous structure embedded within the quadriceps tendon (qt) and separated from the femur (fe) by the patellofemoral joint (arrows). (II.) Fluorescence using digoxigenin- and fluorescein-labelled antisense RNA probes for Sox9 and Scx. At E13.5, cells that express both Sox9 and Scx are observed at the presumable patella location. Scale bars: 200 μm. Modified from [1].

This biochemical theory was first proposed by Eyal *et al*. 2015 [1], as a molecular model for the development of the patella. According to this theory, the patella develops as a bone eminence attached to the distal femur head, where new sox9-positive chondrogenic cells attach to the formed femur distal head (Fig 1II and 1IC) [1]. This new aggregation of chondrogenic cells separates from the preexisting cartilage because of the effect of a remaining joint inducer on the femur articular surface (Fig 1IA). Thus, a new bone embedded within the tendon is formed.

To develop this model, it is necessary to explore both phenomena: the development of the tendon and the eminence formation. The following section describes some of the key events involved in the development of the tendon and bone eminence.

### Tendon & eminence development

**Fig 2:**
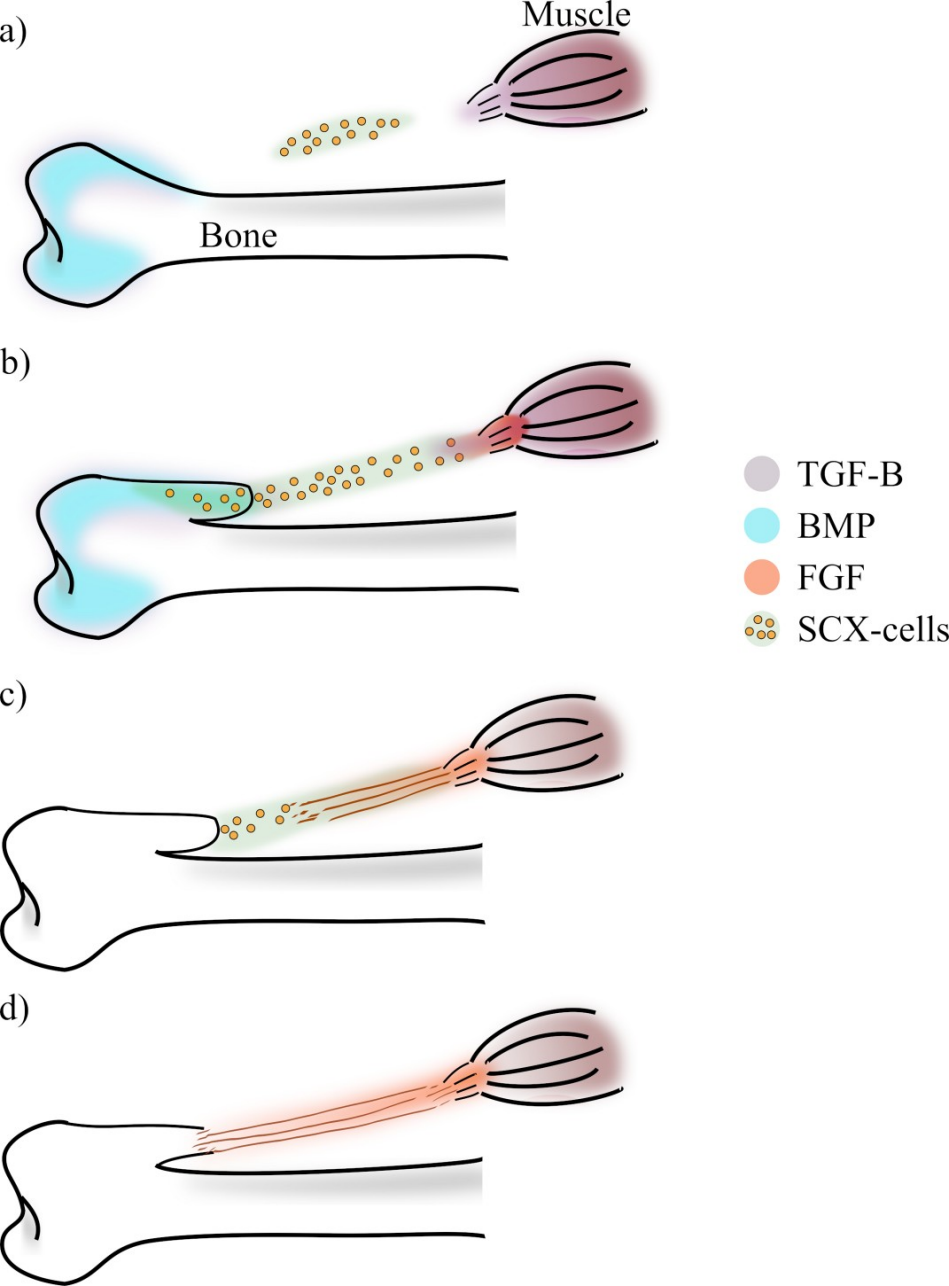
Description of the tendon and eminence development. A) The process starts with the expression of BMP on the bone epiphyses, the bone is still a cartilage mould. Also, the muscle and the epiphyses of the bone express TGF-β. B) The expression of TGF-β attracts and recruits SCX-cells. Those cells that express SCX and BMP start differentiating into chondrocytes. C) The muscle express FGF because its front senses the presence of SCX-cell. FGF induce the differentiation of SCX-cells into tenocytes. d) The formation of the eminence and tendon is complete.

Early events of tendon formation involve the presence of Scx-positive cells (tendon progenitor cells) on the syndetome, a subdomain of the sclerotome (ventromedial compartment of the somite that gives rise to skeletal tissue) [17]. Furthermore, the cells located on the bone heads and muscle ends release Transforming Growth Factor-β (TGF-β). This molecule attracts Scx-expressing cells towards the bone head and muscle end; therefore, the region of this type of cells increases [17] (Fig 2A and 2B).

On the muscle end, the muscle cells perceive the presence of Scx-expressing cells and deliver the Fibroblast Growth Factor (FGF) [18,19]. FGF (that diffuses from the muscle through the Scx-cells domain) promotes the differentiation of Scx-expressing cells into tendon cells (tenocytes). The direction of the gradient of FGF determines the direction of the tendon fibers [18,19] (Fig 2C).

Simultaneously from the bone side, bone morphogenetic proteins (BMPs), which regulate the differentiation of Scx-expressing cells into chondrocytes, diffuse from the bone head [20]. BMP stimulates the Scx-cells to differentiate into chondrocytes. These chondrocytes will later shape the bone eminence where the tendon is attached (Fig 2B and 2C) [20]. Thus, a fully formed tendon is obtained between the bone and the muscle (Fig 2D).

### Computational and mathematical model

**Fig 3:**
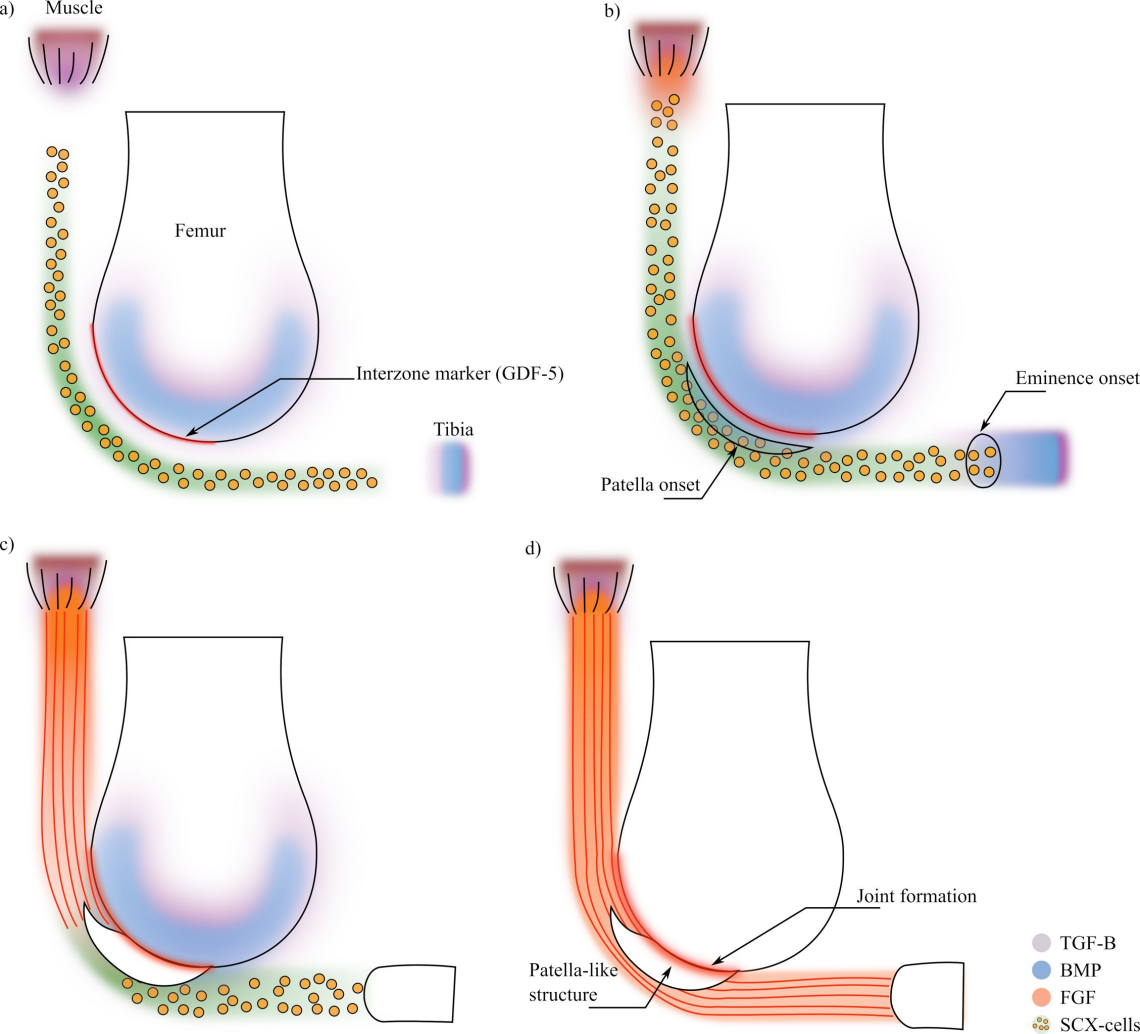
Patella development process based on the theory proposed by Eyal et al. 2015 [1]. a) The process initiates with the SCX-cells necessary for tendon development. The muscle and the bones epiphyses (which in this stage are cartilaginous) express TGF-β which recruit SCX-cells towards the muscle and the bones. Moreover, the bone epiphyses also express BMP factors and some of the remaining interzone marker molecules. b) Those SCX-cells with a concentration of BMP start differentiating into chondrocytes. Also, when the muscle front sense SCX-cells, starts expressing FGF. c) The molecule FGF induce the differentiation of the SCX-cells into tenocytes, whereas the SCX-cells with BMP keep the chondrocyte differentiation, forming the future patella and the tibia eminence. d) The patella forms attached to the cartilaginous femur epiphysis, so a joint formation process starts due to the interzone markers left from earlier processes.

The biochemical model simulates the expression and diffusion of molecular signals during patellar development as proposed by Eyal *et al*. 2015 [1]. It is based on the formation of the quadriceps tendon and patellar tendon (Fig 3) and assumes that some molecules are remnants of the femur-tibia joint formation process [1], such as Growth Differentiation Factor-5 (GDF-5) (Fig 3A) [21–23], which later will induce the joint onset between the patella and the femur.

**Fig 4:**
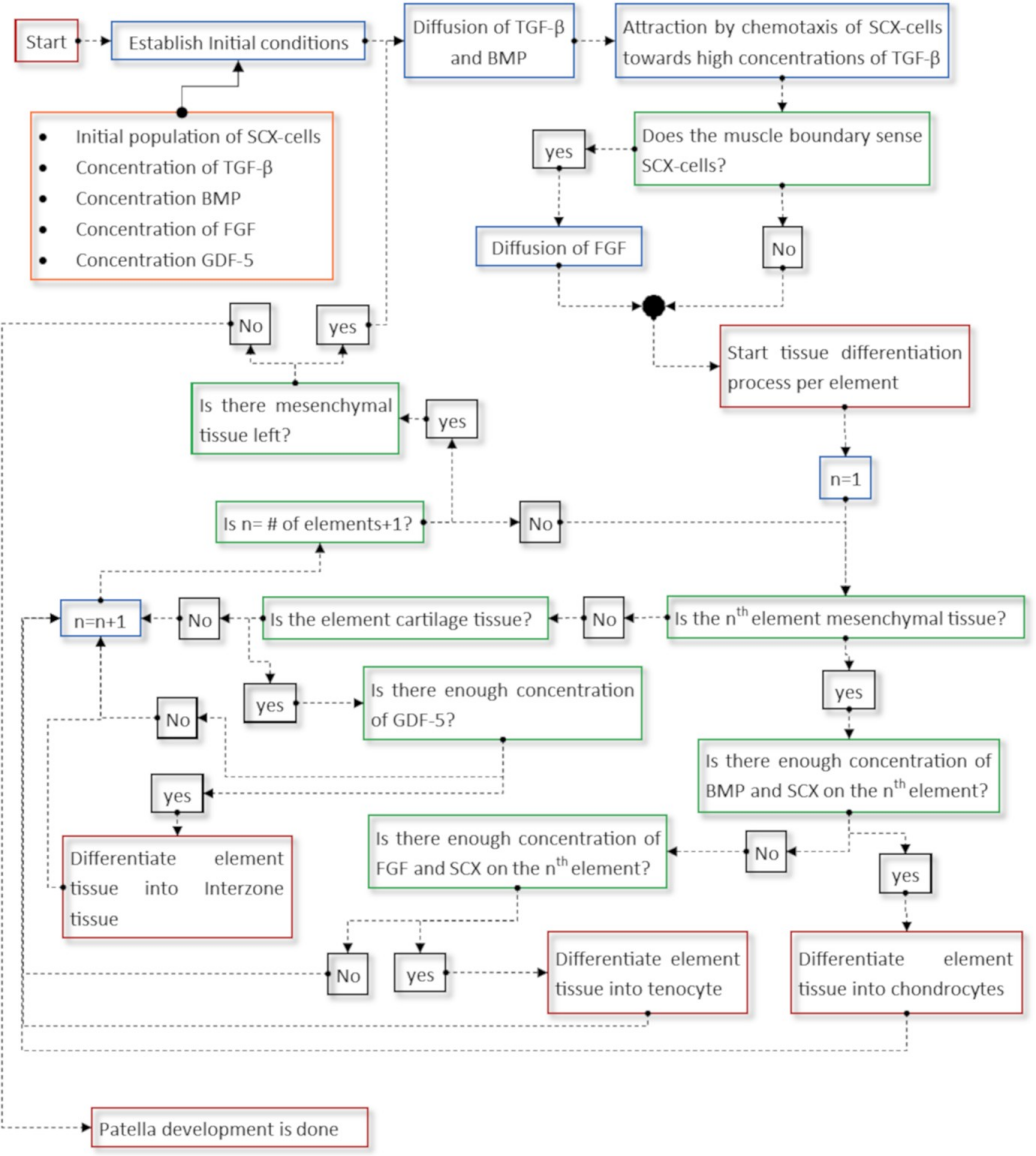
General algorithm that was developed to model the events occurring according to the Theory I.

A general algorithm of this model is shown in Fig 4. The model starts with the initial conditions given by the concentrations of the molecules and the initial domain of Scx-cells (Fig 3A) [17]. Then, TGF-β and BMP diffuse from the muscle and the bone [18,19] and consequently, the Scx-cells are attracted towards high concentrations of TGF-β (Fig 3B). Once the muscle senses the presence of Scx-cells, it expresses FGF (Fig 3B) [18,19]. The differentiation process involves the Scx-cells and the concentrations of FGF and BMP. If there is enough concentration of Scx-cells and BMP, the tissue differentiates into chondrocytes [20], whereas if there is enough concentration of Scx-cells and FGF, the tissue differentiates into tenocytes (tendon tissue) (Fig 3B) [18,19]. At the same time, the remaining interzone marker (in this model named GDF-5) diffuses inside the newly formed cartilage structure (Fig 3C), which later will induce the joint formation between the patella and the femur (Fig 3D) [21–23].

Additionally, it is considered that the flexion of the leg during embryonic stages could influence the patella onset; therefore, this methodology was applied for different leg angles (30°, 45°, 60°, 90°) without the modification of any other parameter.

#### Mathematical model: Molecules

**Sex-cells**. Initially, a domain with Scx-expressing cells is defined where the tendon will be formed [17] (Fig 3). Then, these cells will be attracted towards high concentrations of TGF-β by a chemotaxis process. Therefore, the Scx-cells will move toward the bones and the muscle end (Figs 2 and 3). The concentration of cells (per volume unit) will follow a chemotactic model that will depend on the concentration of the chemoattractant (TGF-β). Eq 1 describes the temporal evolution of Scx-cell density as a function of a diffusion and chemotaxis process.

$$\frac{\partial b}{\partial t}=\nabla .\left[\left(\mu \left(s_{at}\left(x,t\right)\right)\nabla b\left(x,t\right)\right)-\left(\chi \left(s_{at}\left(x,t\right)\right)b\left(x,t\right)\nabla s_{at}\left(x,t\right)\right)\right]+g\left(b\left(x,t\right),s_{at}\left(x,t\right)\right)$$ Eq 1

where *b*(*x*,*t*) is the Scx-cell density population; *s*_*at*_(*x*,*t*) is the concentration of the chemical attractor (TGF-β); *μ*(*s*_*at*_(*x*,*t*)) is the diffusion coefficient of the Scx-cells; *χ*(*s*_*at*_(*x*,*t*)) is the chemotactic coefficient; and *g*(*b*(*x*,*t*),*s*_*at*_(x,t)) is the cell proliferation and death rates.

**TGF-β, BMP, FGF & GDF-5**. The attractant chemical (TGF-β) and the other molecules (BMP, FGF and GDF-5) followed a diffusion model as described in (Eq 2).

$$\frac{\partial s}{\partial t}=D_{i}\nabla^{2}s\left(x,t\right)$$ Eq 2

In this case, *s*(*x*,*t*) is the concentration of the molecule in question (TGF-β, FGF, BMP and GDF-5); *D*_*i*_ is the diffusion coefficient for the molecule *i* with6in the developing tendon or cartilage, with *i* = TGF-β, FGF, BMP and GDF-5. A summary of the diffusion rates is in (S1 Table). It must be noted that these values were obtained by trial and error to match the molecular behavior reported in the literature. Additionally, Table 1 summarizes the relationship between molecules, cells and tissues.

**Table 1:** Relation between molecules and tissue or cell type for the model developed for theory I.

	*Scx-cells*	*Cartilage-patella*	*Cartilage-bone*	*Tendon*	*Muscle*
TGF-β	Attraction towards hi concentrations [18,19]	Low diffusion	Expressed by the tissue [18,19]	—	Expressed by the tissue [18,19]
*FGF*	Differentiation to tenocytes [18,19]	Low diffusion	Low diffusion	Induces the formation of tendon tissue [18,19]	Expressed by the tissue, once the tissue sense Scx-cells [18,19]
*BMP*	Differentiation to chondrocytes [18,19]	Low diffusion	Expressed by the tissue [18,19]	Induces the formation of cartilage (patella) tissue [20]	—
*GDF-5*	—	Induces interzone onset [21–23]	Induces interzone onset [21–23]	—	—

TGF-β: Induced by the bones and the muscle, attracts the Scx-cells towards the bones and the muscle.

FGF: Produced by the muscle, encourages the differentiation of Scx-cells into tenocytes.

BMP: Generated by the bones, incites the differentiation of Scx-cells into chondrocytes.

GDF-5: Joint marker, encourages the split of the forming patella from the femur.

#### Tissue differentiation

The model takes into account that a tissue changes its state when the concentration of a certain molecule achieves a threshold concentration ($b_{c}^{Th},\;S_{BMP}^{Th},\;S_{FGF}^{Th}$ and $S_{GDF-5}^{Th}$) (Table 2). The thresholds were also chosen through an iterative process, since they are not reported in the literature. These values are summarized in the supporting files. Subsequently, a cellular automaton-like model (CA-Like model) was used to regulate the tissue-state based on the concentration of a certain molecule within the tissue.

**Table 2:** Molecule and thresholds involved in the tissue differentiation. The threshold values are specified in the supporting files.

*Original Tissue*	*Molecules*	*New tissue*	*Concentration threshold parameters*
*Mesenchymal*	SCX-cells & BMP	Cartilage	$b_{c}^{Th}\&S_{BMP}^{Th}$
*Mesenchymal*	SCX-cells & FGF	Tendon	$b_{c}^{Th}\&S_{FGF}^{Th}$
*Cartilage*	GDF-5	Interzone	$S_{GDF-5}^{Th}$

Concentrations of Scx-cells and BMP molecules differentiate mesenchymal tissue to chondrocytes (i.e. cartilage tissue), whereas concentrations of Scx-cells and FGF differentiate mesenchymal tissue to tenocytes (i.e. tendon tissue). Additionally, high concentrations of GDF-5 in cartilage tissue form the interzone between the femur and the future patella.

#### Geometry and boundary conditions

**Fig 5:**
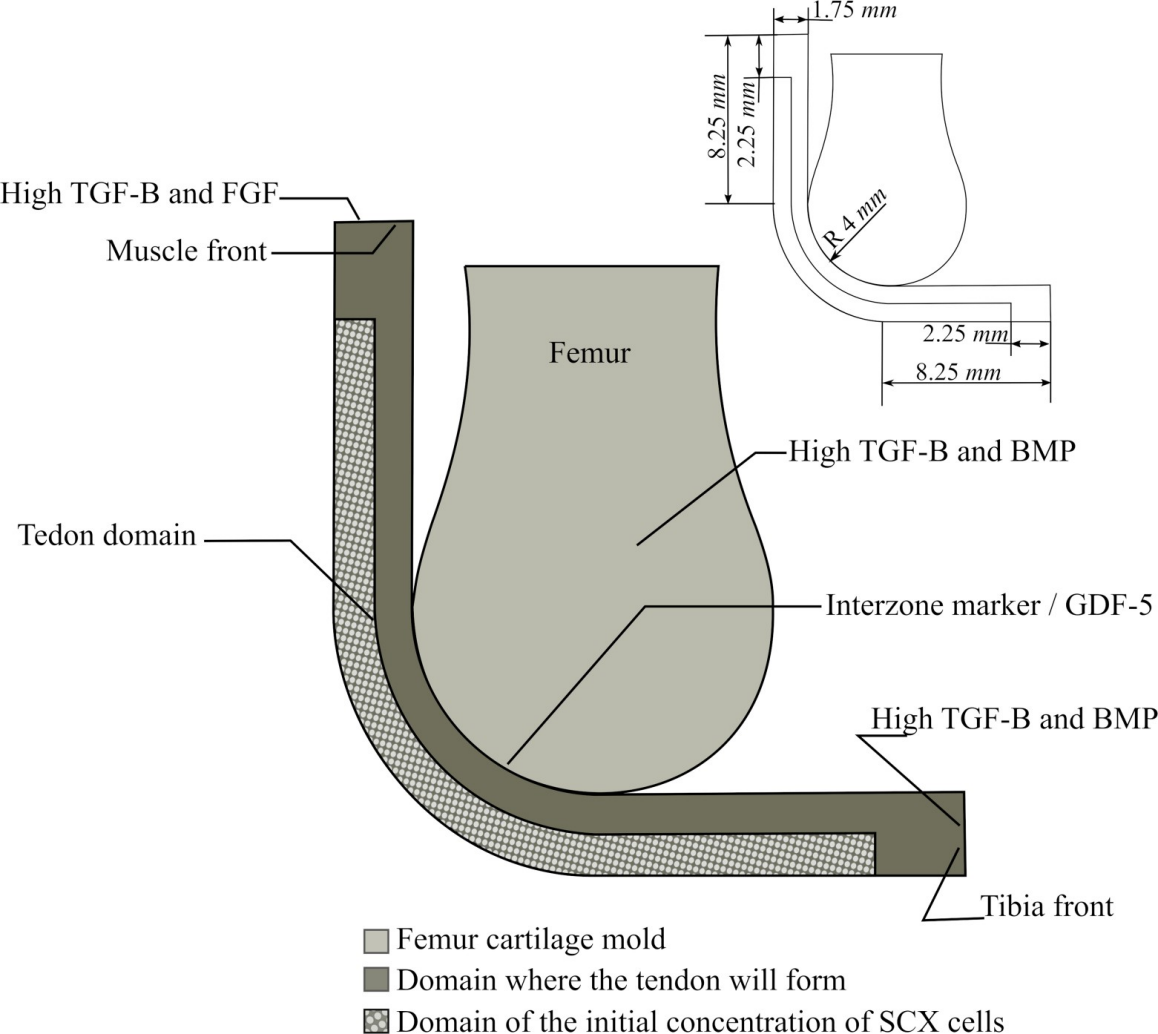
Employed geometry, initial concentrations and mechanical boundary conditions for the model of the theory II.

A simple geometry of the forming knee joint was developed (Fig 5). The geometry is based on the study of Sarin *et al*. [6], where a two-dimensional finite element analysis was developed to determine the stress history of a developing sesamoid. The radius of the condyle was considered as 4 *mm* and the thickness of the tendon as 1.75 *mm* (Fig 5) [24]. Quadrilateral elements were used in the finite element model. The model consisted of three regions: one which represents the femoral cartilage, a second one filled with Scx-cells, and a third one of mesenchymal cells. The tendon domain is formed by the second and third regions. The muscle end has a high concentration of TGF-β and FGF, whereas the tibia end and the femur have high concentrations of TGF-β and BMP [18,19]. In contrast, the interzone marker (GDF-5), that diffuses at a low rate, is on the line shared by the femur cartilage and the domain where the tendon will be (Fig 5).

### Results and discussion

**Fig 6:**
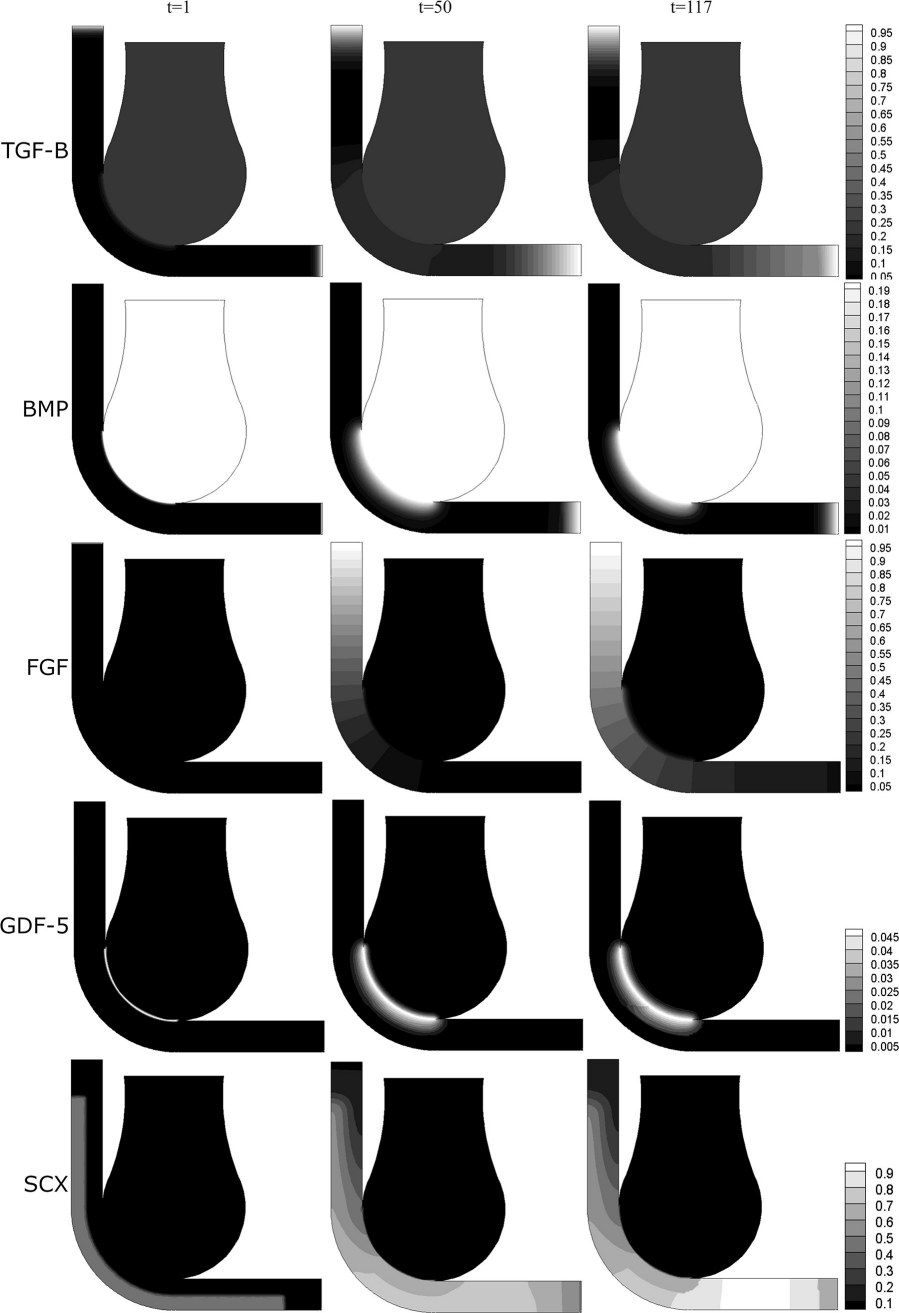
Results: Distribution of the molecules (TGF-β, BMP, FGF, GDF-5). The bar scale shows the concentration of each molecule in ng/ml. Whereas in the bottom of the image is shown the SCX-cells concentration per volume unit involved in the process of patella development; for time t = 0, 7, 42, 50, 60, 117.

**Fig 7:**
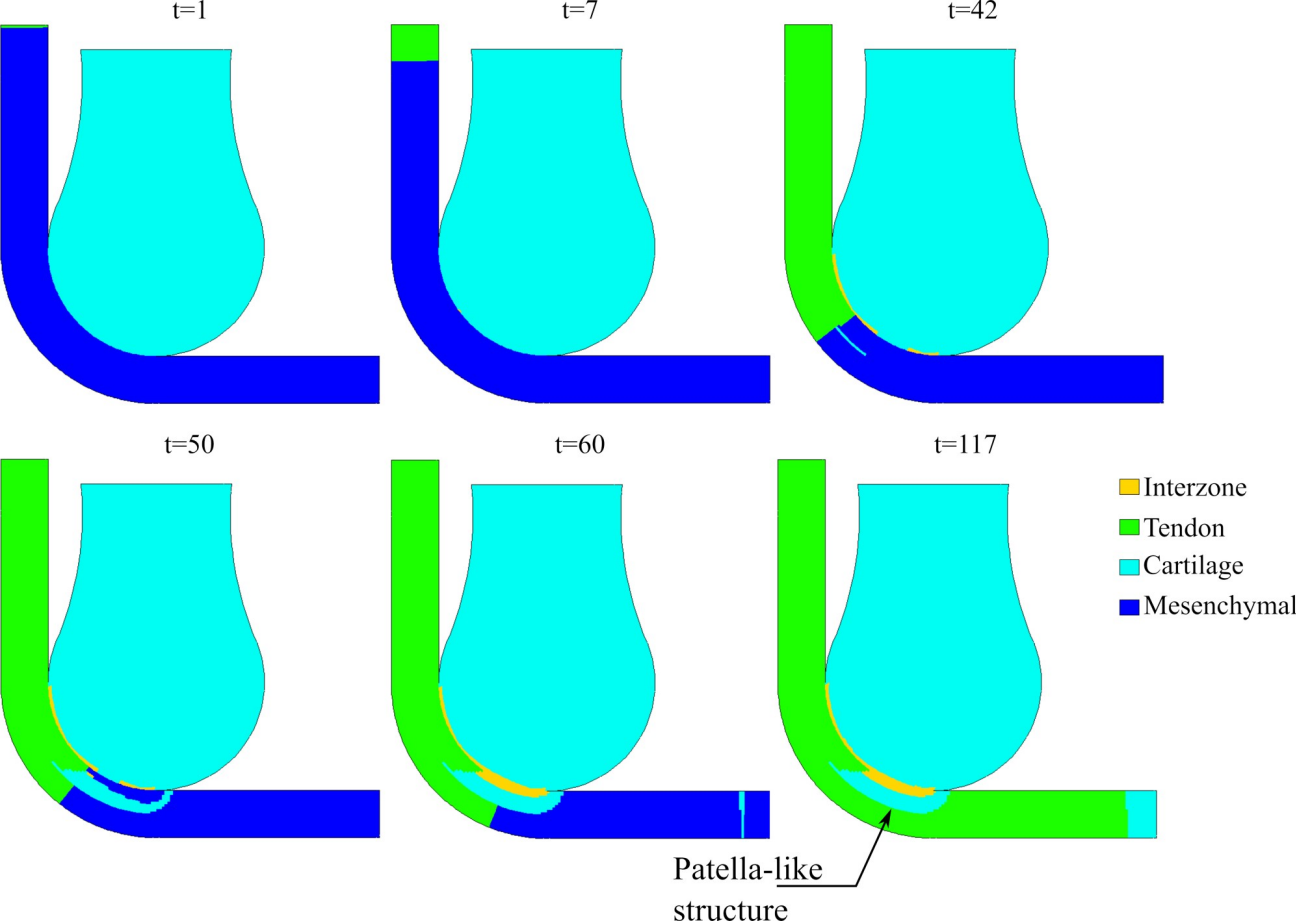
Tissue differentiation through time.

As shown in Fig 6, the molecules TGF-β and BMP diffused and attracted the Scx-expressing cells towards the bones and muscle end. Once the muscle end detected the Scx-cells, the muscle cells expressed FGF; this molecule promoted the differentiation from mesenchymal cells (Scx-cells) into tenocytes, and the FGF gradient settled the direction of the collagen fibers of the tendon (Fig 7). Simultaneously, BMP (which diffused from the femur bone) produced the differentiation from Scx-cells into chondrocytes (Fig 7).

The tendon is formed from the muscle advancing gradually to the tibia (Fig 7, t = 7). Also, a cartilaginous bone eminence developed at a distance from the femoral head, where there was enough concentration of BMP that induced the differentiation from Scx-cells to chondrocytes (Fig 7, t = 42): this was the beginning of the formation of the patella. This cartilaginous structure increased in size towards the femur until both cartilaginous structures (femur anlage and forming patella) merged (Fig 7, t = 60).

At the same time, the tendon continued developing until it reached and embedded the new cartilaginous formation: the patella (Fig 7, t = 50). The result was a developed tendon with a cartilaginous structure, still attached to the femur, and embedded superficially within it (Fig 7, t = 60). Due to the presence of GDF-5, an interzone was formed between the cartilaginous structure (patella-like) and the femur anlage (Fig 7, t = 60). This interzone allowed the detachment of the cartilaginous patella from the femur while still embedded within the tendon (Fig 7, t = 117). The final morphology of the femur and the patella would eventually adapt to mechanical loads due to muscle contractions, with matching articular surfaces. This model simulated only formation of the patella and not morphogenesis.

At some distance from the tibia end, a cartilaginous structure also started forming, where there was enough concentration of BMP and Scx-cells (tibia eminence) (Fig 7, t = 60). The patellar tendon attached to the tibia via this structure (Fig 7, t = 117).

**Fig 8:**
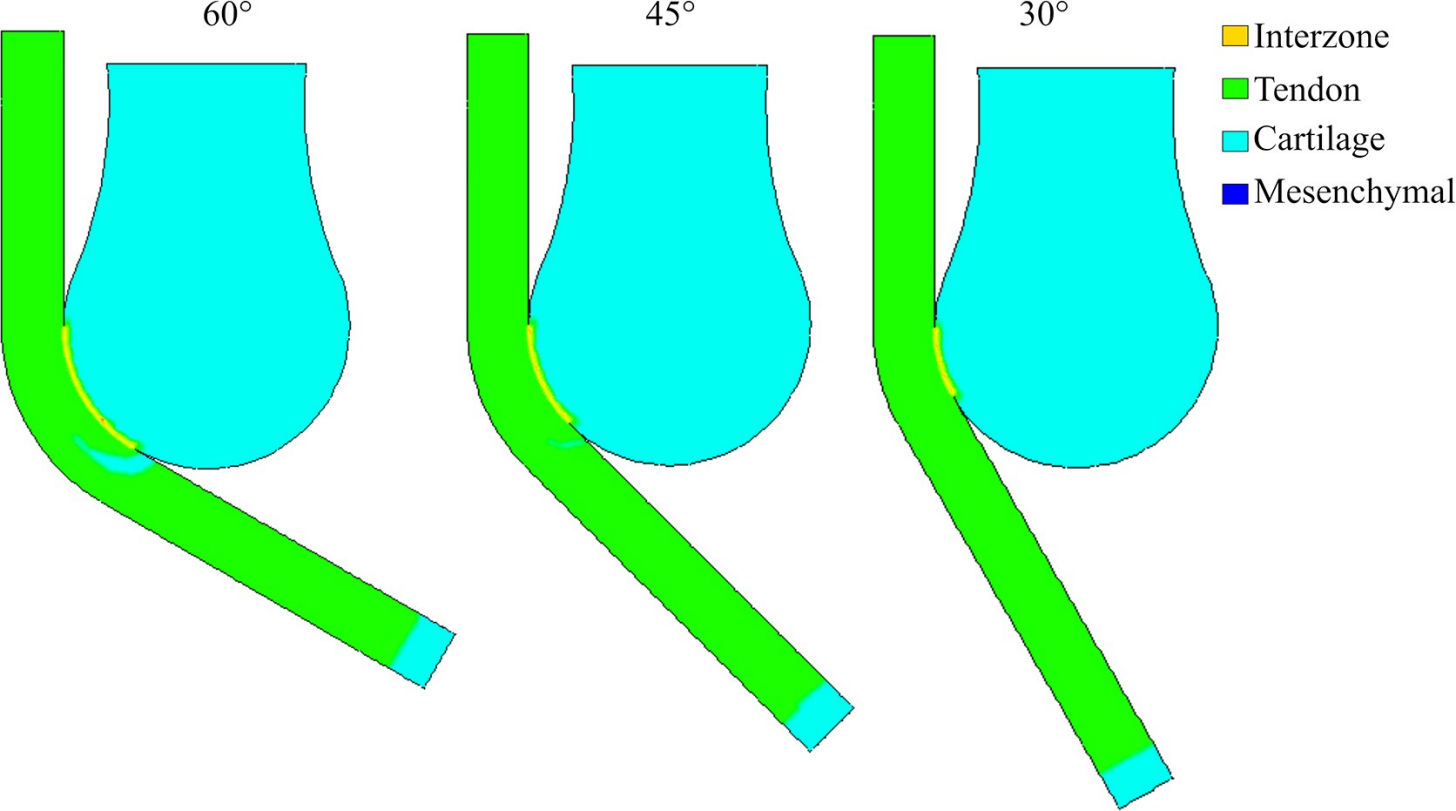
Tissue differentiation for the biochemical model at different angles (60°, 45° and 30°).

This theory was evaluated with different flexion angles of the leg, without modifying any other parameter. Flexion angles of 30°, 45°, 60° and 90° were considered (Fig 8). The range of the angle changed the coincident area between the zone where the tendon will form and the distal head of the femur. The coincident area was smaller for 30°, 45°, and 60°, and larger for 90°. No patella-like structure was achieved with 30° and 45°; only a little incipient patella at 60°; and a complete patella-like structure was obtained with 90°. This outcome was possible since the coincident area is much smaller and proximal with 30°, 45° and 60°. Therefore, there is not enough diffusion of BMP so that the Scx-cells can differentiate into chondrocytes before the tendon develops completely.

The results obtained with this theory for the 90° model were coherent with the histological observations made by Eyal *et al*. 2015 [1]. Additionally, a patella-like structure embedded superficially within the tendon was obtained just by considering the biochemical factors implicated in the tendon and the eminence development (Fig 7). This indicates that the patella onset might be highly influenced by the phenomenon surrounding tendon development. Hence, it might be possible that the patella onset is a consequence of a biochemical process, without any biomechanical influence. This theory could be supported by the fact that the absence of the patella in the knee joint does not affect its functionality [25].

## Theory II: Mechanical theory

The mechanical stimuli play a crucial role in tissue differentiation [7,26]. Although, according to previous studies, when the muscular activity was inhibited in embryos, the patella was smaller than in control animals [27]; therefore, the mechanical conditions of the forming tendons create a favorable environment for the development of the patella [1,5,6,28].

**Fig 9:**
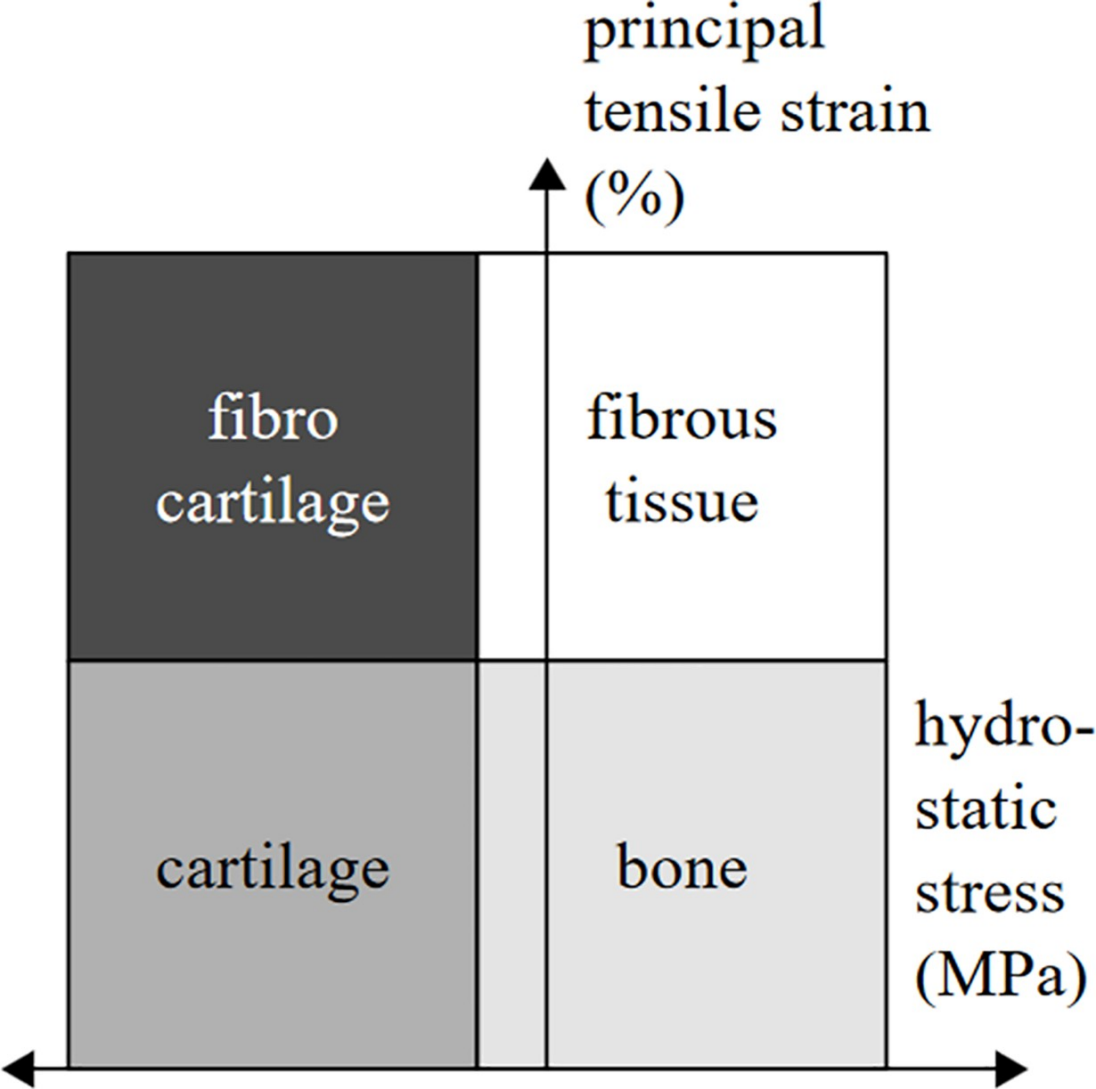
Schematic of the mechanoregulatory model proposed by Carter et al. (1998) *[7]*. Modified from [30].

The tendon is a fibrous tissue composed by bundles of parallel fibers of dense connective tissue, which helps with the role of mediating movement [29]. According to the theory proposed by Carter *et al*. [7], if we have fibrous tissue (tendon) under high compressive hydrostatic stress (with low principal tensile strain), it may differentiate from fibrous tissue to cartilage (Fig 9). This new tissue (within the tendon) will be the sesamoid bone: the patella.

### Mathematical model

Initially, a newly formed fibrous tendon wrapped around the distal end of the femur was assumed. The mechanical load was applied on the quadriceps side of the tendon. The tendon was modelled as a composite material: matrix and fibers. The fibers were modelled as an orthotropic material and they were oriented following the longitudinal axis of the tendon.

The model followed the momentum equation that determines the internal stress es of the body (Eq 3):

∇.σ+b=0 Eq 3

where ***σ*** is the stress tensor, and ***b*** is the body forces. The stresses and strains were related through the constitutive equation, which in general from is given by (Eq 4):

σ=Dε Eq 4

where ***D*** is the matrix of elastic constants, and ***ε*** is the strain tensor. Since the tendon was composed of a matrix and fibers, then, the relationship between stress and strain is given by (Eq 5):

$$\sigma =\sigma_{m}+\sigma_{f}=\left(D_{m}+D_{f}\right)\varepsilon =D_{m}Bu+D_{f}Bu$$ Eq 5

where ***D***_***m***_ and ***D***_***f***_ are the matrix of elastic constants for the matrix and the fibers, respectively. The matrix was considered as an isotropic material; therefore, the term ***D***_***m***_ is defined as (Eq 6)

$$D_{m}=\frac{E}{1-v^{2}}\left[\begin{array}{ccc} 1 & v & 0\\ v & 1 & 0\\ 0 & 0 & \left(1-v\right)/2 \end{array}\right]$$ Eq 6

Whereas ***D***_***f***_, for a link for fiber oriented on the horizontal axis, is defined as (Eq 7):

$$D_{f}B=\left[\begin{array}{cccc} 1 & 0 & -1 & 0\\ 0 & 0 & 0 & 0\\ -1 & 0 & 1 & 0\\ 0 & 0 & 0 & 0 \end{array}\right]$$ Eq 7

**Fig 10:**
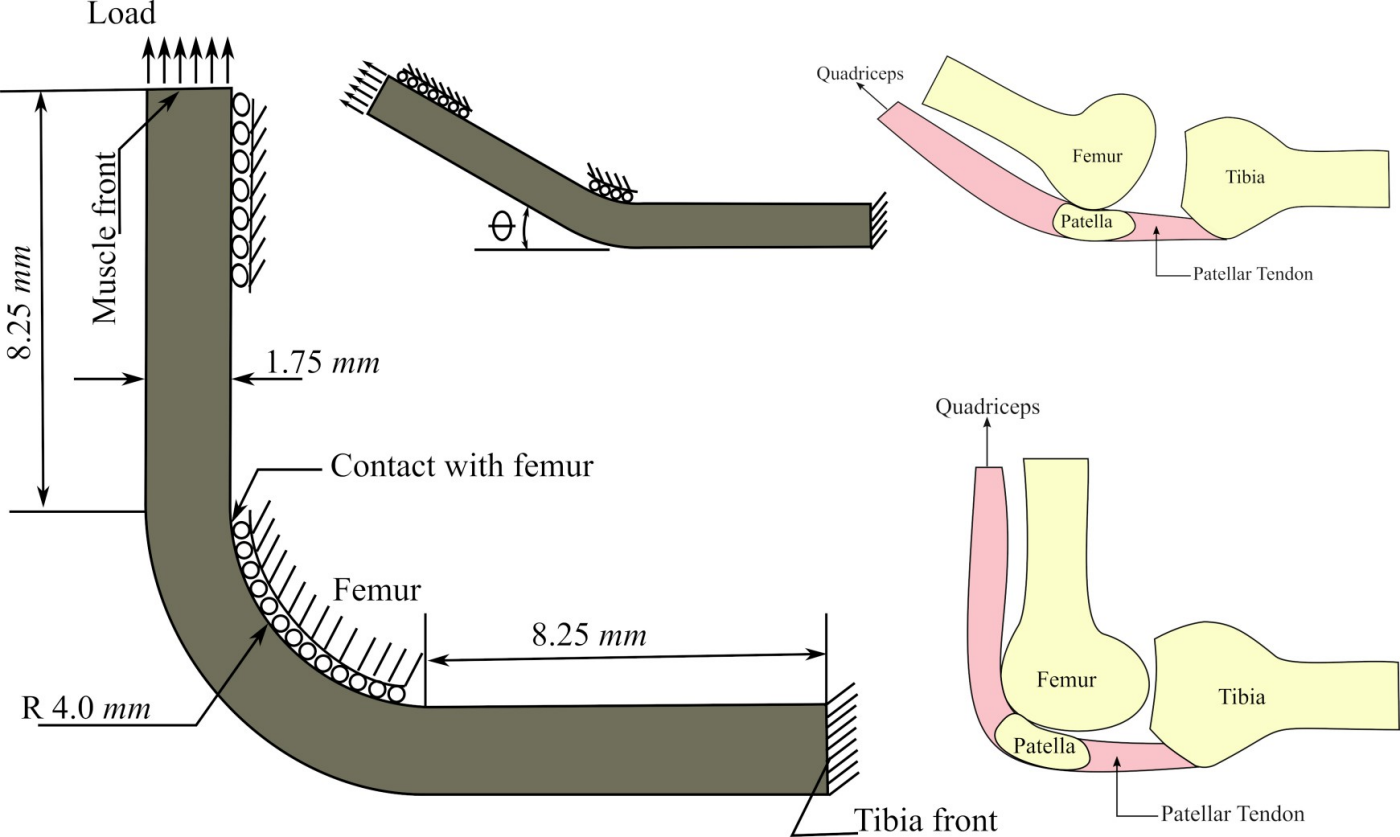
Employed geometry, initial concentrations and mechanical boundary conditions for the model of the theory I.

The stress inside the tendon was evaluated at different flexion angles: θ = 110°, 90°, 60°, 45°, and 30° (Fig 10). In order to facilitate the comparison of results, each case (θ = 110°, 90°, 60°, 45°, and 30°) had the same number of finite elements; in other words, there was a correspondence of finite elements between cases.

#### Geometry and boundary conditions

The geometry of this model was a simplified version of a knee joint in formation (Fig 10) based on similar works [6,31]. The geometry considers a tendon wrapped around the femur distal head. The thickness of the tendon was 1.75 *mm* and the radius of the bone was 4.0 *mm* [24].

The mechanical load was applied on the tendon as 1*mm* displacement, simulating the contraction force of the quadriceps, whereas the other end of the tendon (attachment with the tibia) was fixed (Fig 10). The nodes on the line in contact with the femur could only move through the contact line (Fig 10).

#### Material properties and tissue differentiation

The differentiation from tendon tissue to cartilage was assumed as a function of hydrostatic stress and tensile strain following Carter *et al*. [7]. When the hydrostatic stress and the principal tensile strain on the fibrous tissue (tendon) reached a specific threshold—$\sigma_{hyd}^{cart}$ (supporting files)- the tissue differentiated into cartilage. Table 3 summarizes the mechanical properties of the biological tissues used in this work. All the properties were obtained from the literature [6,32]. The properties of the tendon depended on the direction of the tendon collagen fiber.

**Table 3:** Tissue properties.

*Tissue*	*Poisson*	*Young’s modulus MPa*	*Reference*
*Cartilage*	0.497	6.1	[33]
*Tendon (matrix)*	0.4	6.1	[6]
*Tendon (fiber)*	0.4	800	[6]

### Results and discussion

**Fig 11:**
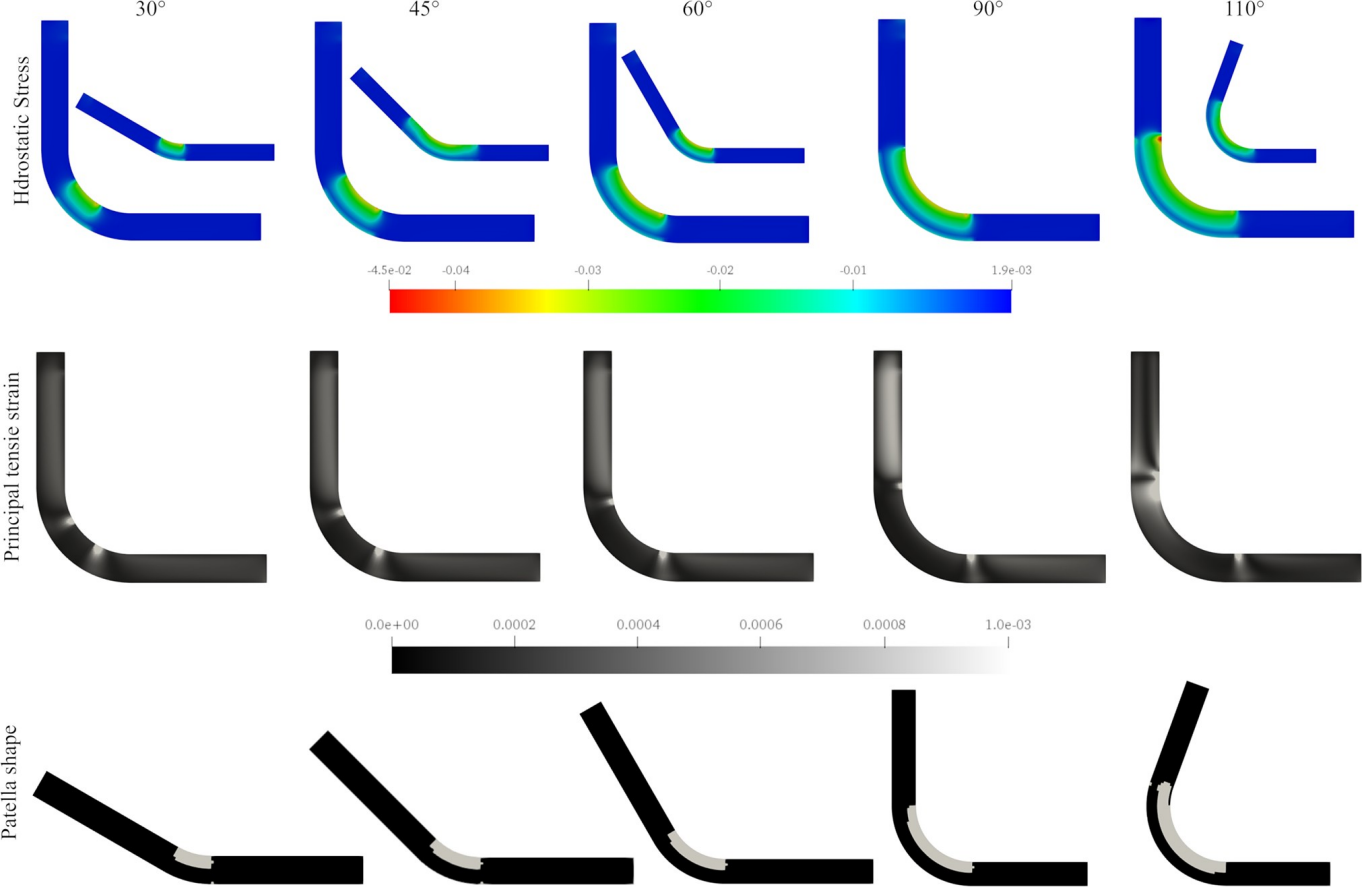
Obtained stress, tensile strain and patella shape. Up: Hydrostatic stress distribution on the tendon at different angles 30°, 45°, 60°, 90° and 110°. The value of the hydrostatic pressure was projected on the 90° domain for comparison. Middle: principal tensile strain for 30°, 45°, 60°, 90° and 110°. Bottom: the obtained patella shape, where the elements that have the hydrostatic stress above the tissue differentiation threshold $\sigma_{hyd}^{cart}$.

This theory posits that the patella bone is formed due to high hydrostatic stress and low tensile strain on specific regions of a fibrous tissue, such as the tendon. The results of all angles of flexion of the leg were projected on the domain at 90° to facilitate comparison of the stress distributions (Fig 11).

Considering that the understanding of the embryo movement is limited, three different scenarios were modelled in which the effect of each leg angle was averaged. These scenarios were designed in order to differentially weigh the contribution of each angle to represent the time spent in that angle. The weightings for the angles of 30°, 45°, 60°, 90° and 110° for the three cases were as follows: 1) 5%, 10%, 15%, 25% and 45% (weighting high flexion), 2) 60%, 25%, 9%, 5%, and 1% (weighting low flexion angles); 3) 1%, 5%, 9%, 25% and 60% (heavily weighting high flexion).

**Fig 12:**
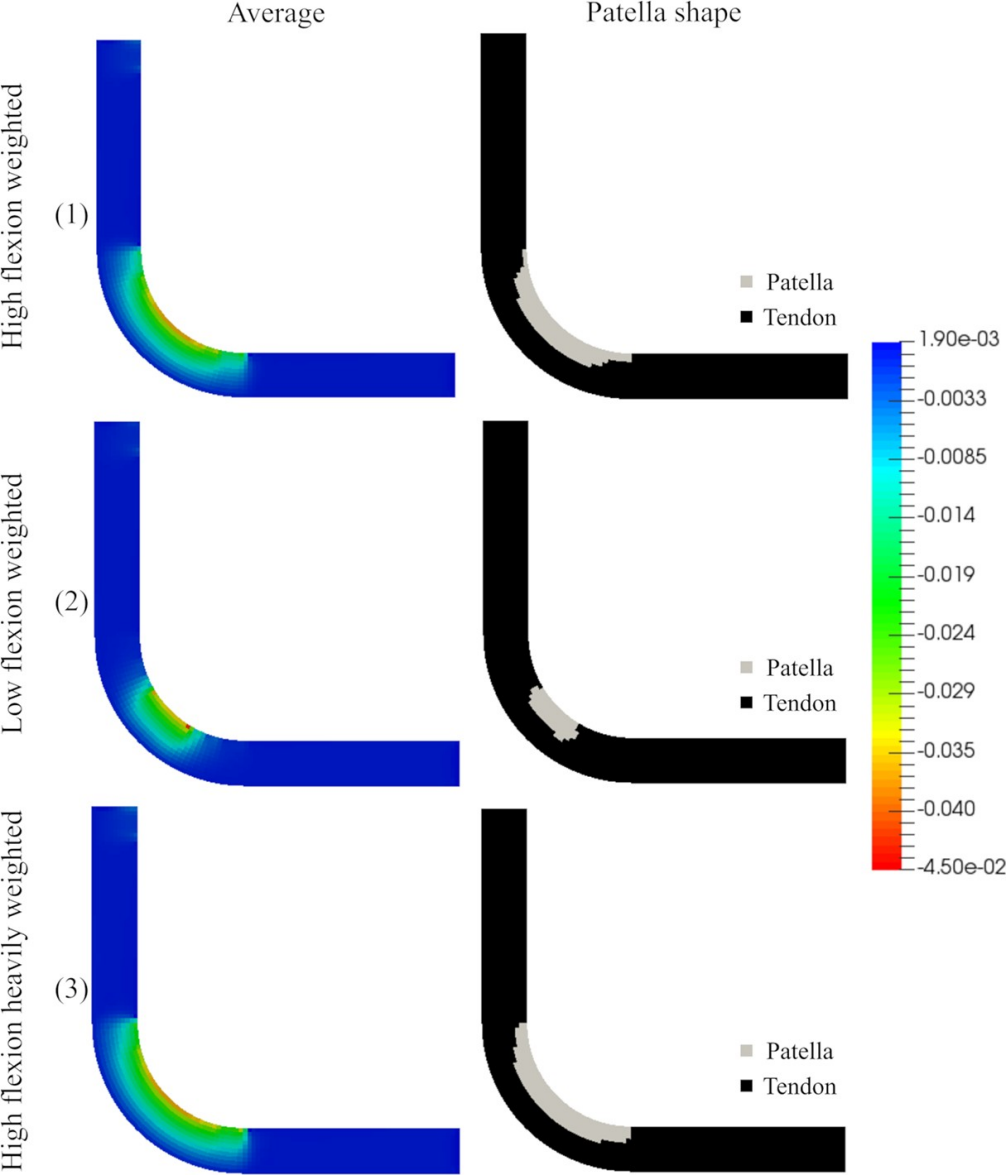
Obtained average hydrostatic stress and patella shape. Left: Average hydrostatic stress for the three analyzed cases- 1) 5%, 10%, 15%, 25% and 45% (weighting high flexion), 2) 60%, 25%, 9%, 5%, and 1% (weighting low flexion angles); 3) 1%, 5%, 9%, 25% and 60% (heavily weighting high flexion) for the 30°, 45°, 60°, and 90° respectively. Right: Patella shape: where the elements that have the hydrostatic stress above the tissue differentiation threshold $\sigma_{hyd}^{cart}$.

The elements, which had a compression stress above $\sigma_{hyd}^{cart}$, were differentiated into cartilage (Fig 12). In each case the size and shape of the patella were different; the smallest patella occurred when weighting the smallest angles (Fig 12).

The results obtained with this model also showed the development of a patella-like structure embedded within the tendon. The size of the structure depended on the flexion angle; in other words, the angle determined the mechanical load (hydrostatic pressure) in the tendon. This may imply that the mechanical conditions that surround the newly formed tendon might influence the patella development. For instance, studies have noticed that several years after the excision of the patella, some fibrocartilage or even bony islands appear in the former site of the patella [25,34,35]. This could indicate that the wrapping (mechanical environment) of the tendon over the distal femoral head generates the necessary mechanical conditions for the patella onset.

## Theory III: Topological optimization

### Mathematical model

This theory is also based on mechanical stimuli and its influence on tissue differentiation. It is founded on the fact that tissue adapts to their stress and strain environment; therefore, Topological Optimization (TO) might be suitable to explain this adaptation. TO can be applied in tissue remodelling, since it iteratively redistributes the material in a design domain determining an optimal material arrangement or tissue type [11]. Specifically, TO allocates denser material (cartilage) to regions under relatively high strain energy and allocates a less dense material (tendon) to zones with a low strain energy. This adaptation process can be characterized as a self-enhancing system [8], with the objective of minimizing tendon strain. Therefore, the articular surface wear will be reduced due to the low relative movement between the distal femoral head joint surface and the tendon.

As an initial condition, it was considered that the tendon was already formed and loaded. A two-dimensional FEA analysis based on the algorithm proposed on Sigmund [36], was performed. The algorithm is founded on the “power-law approach” or SIMP approach (Solid Isotropic Material with Penalization) [36]. This approach assumes that the material properties are constant within each element of the design domain, whereas the relative material densities of the elements are the variables [36]. The density is usually considered as a design variable, so it could take values between 0 and 1, with 0 representing void and 1 representing solid [37]. Thus, the relation between the elastic modulus and the relative material density is given in Eq 8:

$$E\left(x\right)=\rho {\left(x\right)}^{p}E^{o}$$ Eq 8

where *E*^*o*^ is the Young’s modulus of the material in solid state, when *ρ* = 1. In addition, the penalization power parameter is p = 3 as recommended by Sigmund [36].

The aim of this optimization process is to minimize the strain energy in order to find the relative material densities of the elements (in a design domain). Then, the objective function and constraints can be expressed as follows:

$$\min_{\rho }C\left(\rho ,u\right)=U^{T}F$$ Eq 9

$$s.t.:\begin{array}{c} K(\rho )u=F\\ \frac{V(\rho )}{V_{o}}=f\\ 0<\rho_{min}\leq \rho (x)\leq 1 \end{array}\}$$

where *C* is compliance, *ρ* is density, **F** is the load vector, ***u*** is the global displacement vector, *V*_*o*_ is the initial domain (domain constraint) and *f* is the volume fraction. The density was relaxed to have any value from 0 to 1, being the lower bound non-zero to avoid singularities.

The global stiffness matrix in Eq 9 is calculated by summing up all the elemental stiffness matrices, which depend on the elemental value of the density *ρ*_*e*_ Eq 10:

$$K\left(\rho \right)=\sum_{e=1}^{Nel}K_{e}\left(\rho_{e}\right)=\sum_{e=1}^{Nel}\int_{\Omega_{e}}B^{T}D\left(\rho_{e}\right)Bd\Omega$$ Eq 10

where ***K***_***e***_(*ρ*_*e*_) is the stiffness matrix of the element, ***B*** is the shape function derivatives, and ***D***(***ρ***_***e***_) is the constitutive matrix which depends on the material density. Sensitivities of the objective function and volume constraint with respect to *ρ*_*e*_ are calculated as follows:

$$\frac{\partial C}{\partial \rho_{e}}=-u_{e}^{T}\frac{\partial K_{e}}{\partial \rho_{e}}u_{e}=-p\rho_{e}^{p-1}u_{e}^{T}K_{e}^{o}u_{e}$$ Eq 11

$$\frac{\partial V}{\partial \rho_{e}}=\int_{\Omega_{e}}dV$$ Eq 12

A heuristic updating scheme for the design variable is formulated as:

$$\rho_{e}^{new}=\left\{ \begin{array}{l} \max\left(\rho_{min},\rho_{e}-m\right)\;if\;\rho_{e}B_{e}^{\eta }\leq \max\left(\rho_{min},\rho_{e}-m\right),\\ \rho_{e}B_{e}^{\eta }\;if\;\max\left(\rho_{min},\rho_{e}-m\right)<\rho_{e}B_{e}^{\eta }<\min\left(1,\rho_{e}+m\right),\\ \min\left(1,\rho_{e}+m\right)\;if\;\min\left(1,\rho_{e}-m\right)\leq \rho_{e}B_{e}^{\eta }, \end{array}\right.$$ Eq 13

where *m* is a positive moving limit, *η* is the numerical damping coefficient and *B*_*e*_ is the optimality condition which is calculated as:

$$B_{e}=\frac{\frac{\partial C}{\partial \rho_{e}}}{\lambda \frac{\partial V}{\partial \rho_{e}}}$$ Eq 14

#### Geometry and boundary conditions

The same geometry and boundary conditions of Theory II were used in this model.

#### Material properties

The tissue properties of the initial domain (tendon and fibers) are summarized in (Table 3). The properties of the tendon depended on the direction of the tendon collagen fibers.

### Results and discussion

**Fig 13:**
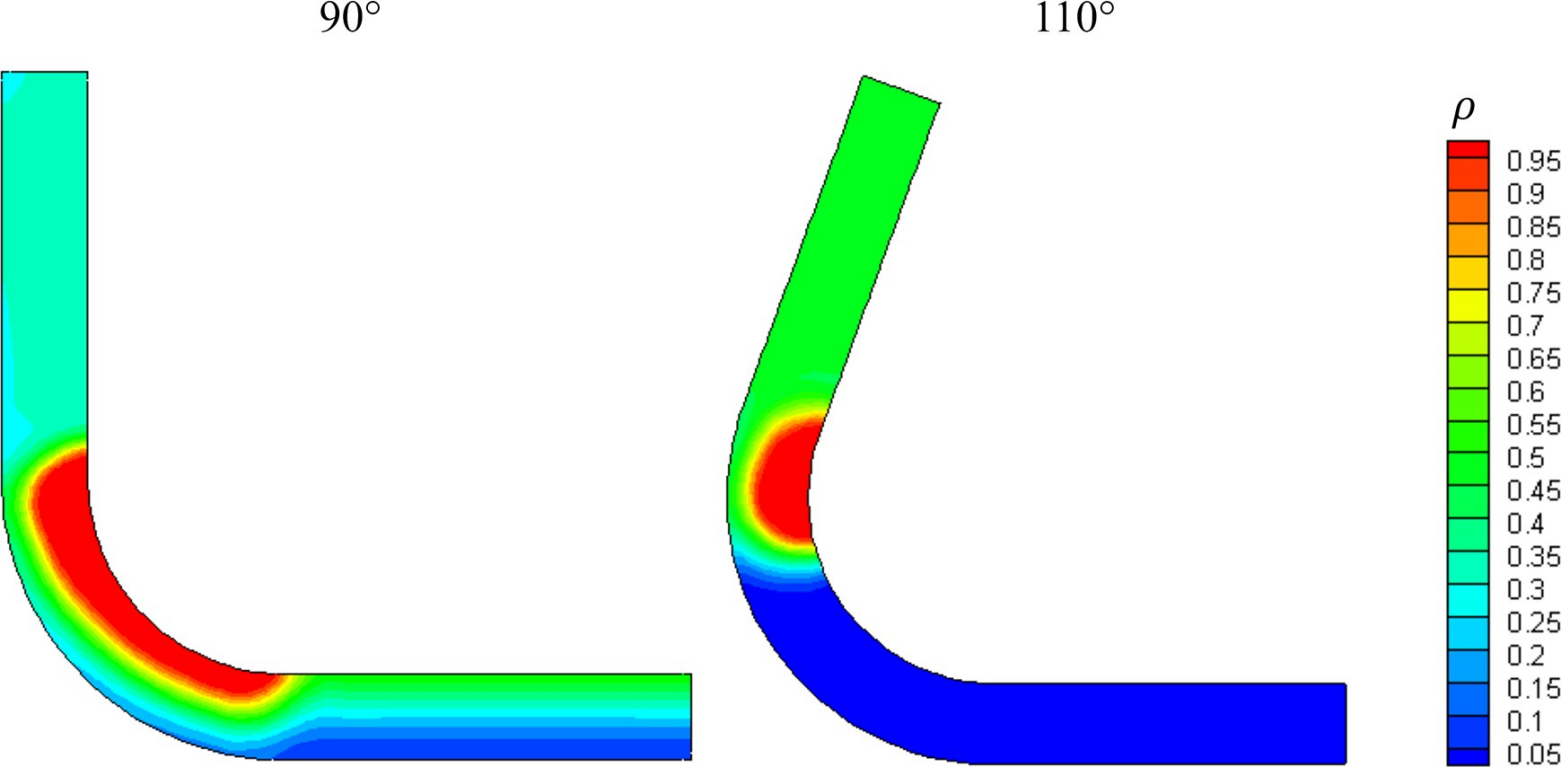
Density values for the patellar tendon at 90° and 110°.

In this third theory, the appearance of the patella is due to a topological optimization process in the recently formed patellar tendon. Different angles were tested for this model; however, we only obtained coherent results (formed patella) with 90° and 110°. For these angles, we observed a high-density zone close to the usual patella position. Specifically, for the 90° case, the shape of the high-density zone is more consistent with reality (Fig 13). This suggests that the mechanical environment that surrounds the tendon may affect the patella and its development.

Some studies have uncovered that quite a long while after the extraction of the patella a, islands of fibrocartilage, or even bone show up in the previous site of the patella. [34,35]. These islands might be the result of the optimization process that the human body undergoes due to the abnormal conditions after the patella excision. The strain energy reduces in this process, which allows the tendon to have small displacements and deformations.

## Numerical solution of the equations for the three theories

All the equations of the three theories were solved with finite element methods as implemented in a user Subroutine in Fortran and solved with ABAQUS 6.10 (Dassault Systemes USA, Waltham, MA). Both mesh and the time step were refined until further refinement no longer yielded noticeable improvements in all models.

## General discussion

We developed three models to simulate the process of patella formation according to three different theories. The aim was to evaluate, individually, the potential influence of each of these theories in the patella onset. The first theory considers the biochemical interactions present during the patellar tendon formation, while the second and third theories are based on the mechanical stimuli that the newly developed patellar tendon goes through. All models used a simplified knee geometry, considering both the tendon and the femur for the biochemical-based theory (Fig 5), and only the tendon for the mechanical-based theories; the femur was assumed as a rigid body (Fig 10).

The biochemical model is a conceptual model of the development of the patella based on the theory proposed by Eyal et al. 2015 [1], where the patella initially forms as a bone eminence, involving Scx-cells whose differentiation is regulated by TGF-β and BMP-4. The separation of the patella from the femur is regulated through the interzone molecule GDF-5. The simulated molecular distribution agrees with that shown in Eyal et al. 2015 [1], where at the end a patella-like structure embedded superficially within the tendon was obtained. We assumed the diffusion rates of the molecules based on an iterative process since they are not reported in the literature and are particularly difficult to measure.

Additionally, we also simulated the biochemical model when the leg was extended at different angles (30°, 45°, 60°, 90°). The latter was to establish how the position of the leg affected the development of the patella, in the case that only biochemical factors influenced the formation of this sesamoid bone. When the angles of the leg were modified to 30°, 45° or 60°, the region through which BMP diffused from the femur distal end to the tendon region was smaller. Consequently, the patella obtained was either not significant or null (Fig 8). This may be evidence, according to this theory, that having an angle close to 90° is necessary to form the patella.

On the other hand, the first mechanical model is based on the theories that state that mechanical stimuli play a crucial role in tissue differentiation. Particularly, this model is built upon Carter’s theory, in which hydrostatic compression and low principal strain are stimuli for fibrous tissue differentiation into cartilage. It was assumed that the constant movements of the fetus (distinct knee angles) stimulates the concentration of high compression loads on some areas of the newly formed tendon. The latter induces the differentiation of the tendon cells into cartilage, on those high hydrostatic stress zones. Therefore, a cartilage structure is formed embedded within the tendon, which later will ossify and become the patella bone.

Following this last theory (the mechano-differentiation model), a patella-like structure was obtained. When different flexion angles of the leg were evaluated, we observed an influence on the size of the patella (Fig 11). Additionally, a patella-like structure was obtained when the hydrostatic stress was averaged considering all the angles. A different predominant angle for each case was evaluated: weighting high flexion, weighting low flexion angles and heavily weighting high flexion. If the predominant angle was large, a big patella was obtained, whereas if the predominant angle was small, a short patella was obtained (Fig 12).

The last evaluated theory was based on the application of topological optimization, which is also feasible to explain the onset of sesamoid bones such as the patella. In this case, we assume that the tissue can adapt to its mechanical environment in a way that its strain energy is reduced. Different angles were also evaluated. However, the outcome was not as expected for smaller angles: no patella-like structure was obtained. Nevertheless, a patella-like structure was achieved with angles of 90° and 110°. The shape of the obtained patella is remarkably similar, especially with an angle of 90°. The results of this model imply that the patella might allow reducing the strain energy and, consequently, the displacements and deformations of the tendon. These results also imply that the patella and the femur articular surface have a low relative movement. This is due to the low deformation of the tendon, protecting somehow the femur articular surface from wear.

A patella-like structure was obtained for most of the knee flexion angles in all the theories we evaluated. Our results show that tissue remodelling and adaption, based on Carter’s theory or on topological optimization, could answer the patella onset. The shape, position and size of the patella would depend on the flexion angle of the leg, and the time that the leg spends on each position, obtaining a larger-sized patella compared to the biochemical approach. However, there is not much evidence in the literature that can support the second and third theories. Some studies have shown that while limitation of movement (through drugs) on embryos affects the formation of the patella, it develops anyway, although small in size [27]. Therefore, the mechanical load may not be necessary for the appearance of the patella, but it may be necessary for its morphogenesis and maintenance. Hence, molecular factors and their interactions trigger the formation of the patella, as evidenced by molecular expression analyzed in histological slides [1]. These factors applied to a computational model were consistent with the results obtained. However, since only the limitation of the movement does not guarantee that there are no hydrostatic stresses on the tendon, more tests should be run in which only biochemical factors influence the development of the tendon and the patella. It is also possible that these mechanisms are redundant and that both influence the patella onset.

This article proposes a simplified mathematical model of the regulatory mechanisms that might influence the formation of the patella bone. Each theory was evaluated separately to observe its outcome and the likelihood of its influence on the patella onset. The results obtained were consistent, and patella-like structures were obtained in most cases. Nevertheless, the literature suggests that the patella onset could be triggered by biochemical factors during tendon development [1]; according to our results, this approach does lead to a patella-like structure. Also, it is certain that the mechanical environment must affect the patella development. However, this environment might affect it mostly after the tendon is formed by helping the patella to obtain its final shape and maintain its structure.

This work is a first approximation on understanding the process of the development of the patella. It should be considered that these models had several simplifications, such as that they were all two-dimensional models, that the geometry was a simplified knee joint, that all the materials were modelled as linear elastic, and that the molecular concentrations, since they are difficult to measure and there are no reports in literature, were established through an iterative process. Even though some simplifications were assumed, we obtained results that might explain the onset of the patella and would help in the proposition of new points of view that might explain the patella onset. Furthermore, these models could provide new insight and guidelines of experimentation, and of course, new mathematical models as well. However, a combination of the theories evaluated in this study is suggested in future works, so that the patella onset is determined due to biochemical factors, and thereafter the mechanical loads may regulate its shape and maintenance. The exact instant and way that mechanical loads affect the patella development should be an issue to evaluate in further models, as well as the dynamic movement of the knee during the development.

## Supporting information

S1 Table Values of the coefficients employed on the model of theory I and theory II. (DOCX)

## References

[1] Eyal S, Blitz E, Shwartz Y, Akiyama H, Ronen S, Zelzer E. On the development of the patella. Development. 2015; 1–9. 10.1242/dev.119727 25926361 10.1242/dev.121970

[2] Sutton FS, Thompson CH, Lipke J, Kettelkamp DB. The effect of patellectomy on knee function. J Bone Joint Surg Am. 1976;58: 537–540. Available: http://www.ncbi.nlm.nih.gov/pubmed/1270472 1270472

[3] Schindler OS, Scott WN. Basic kinematics and biomechanics of the patello-femoral joint Part 1: The native patella. Acta Orthop Belg. 2011;77: 421–431. 21954748

[4] Mottershead S. Sesamoid bones and cartilages: An enquiry into their function Clin Anat. Wiley Subscription Services, Inc., A Wiley Company; 1988;1: 59–62. 10.1002/ca.980010110

[5] Howale DS, Patel ZK. Hypothesis: Morphology & Development of Patella. 2013;3: 1–5.

[6] Sarin V, Carter D. Mechanobiology and joint conformity regulate endochondral ossification of sesamoids. J Orthop Res. 2000;18: 706–712. 10.1002/jor.1100180505 11117290 10.1002/jor.1100180505

[7] Carter DR, Beaupré GS, Giori NJ, Helms JA. Mechanobiology of skeletal regeneration. Clin Orthop Relat Res. 1998; S41–55. doi: Non-programmatic 9917625 10.1097/00003086-199810001-00006

[8] Jang IG, Kim IY. Application of design space optimization to bone remodeling simulation of trabecular architecture in human proximal femur for higher computational efficiency. Finite Elem Anal Des. Elsevier; 2010;46: 311–319. 10.1016/j.finel.2009.11.003

[9] Cai K, Luo Z, Wang Y. Topology optimization for human proximal femur considering bi-modulus behavior of cortical bones. Springer Proc Math Stat. 2015;95: 263–270. 10.1007/978-3-319-08377-3_26

[10] Coelho PG, Fernandes PR, Rodrigues HC, Cardoso JB, Guedes JM. Numerical modeling of bone tissue adaptation-A hierarchical approach for bone apparent density and trabecular structure. J Biomech. 2009;42: 830–837. 10.1016/j.jbiomech.2009.01.020 19269639 10.1016/j.jbiomech.2009.01.020

[11] Jang IG, Kim IY. Computational study of Wolff’s law with trabecular architecture in the human proximal femur using topology optimization. J Biomech. 2008;41: 2353–2361. 10.1016/j.jbiomech.2008.05.037 18667206 10.1016/j.jbiomech.2008.05.037

[12] Boyle C, Kim IY. Three-dimensional micro-level computational study of Wolff’s law via trabecular bone remodeling in the human proximal femur using design space topology optimization. J Biomech. Elsevier; 2011;44: 935–942. 10.1016/j.jbiomech.2010.11.029 21159341 10.1016/j.jbiomech.2010.11.029

[13] Chang CL, Chen CS, Huang CH, Hsu ML. Finite element analysis of the dental implant using a topology optimization method. Med Eng Phys. Institute of Physics and Engineering in Medicine; 2012;34: 999–1008. 10.1016/j.medengphy.2012.06.004 22770748 10.1016/j.medengphy.2012.06.004

[14] Kang H, Lin CY, Hollister SJ. Topology optimization of three dimensional tissue engineering scaffold architectures for prescribed bulk modulus and diffusivity. Struct Multidiscip Optim. 2010;42: 633–644. 10.1007/s00158-010-0508-8 10.1007/s00158-010-0508-8 PMC7413610 32774195

[15] Sutradhar A, Paulino GH, Miller MJ, Nguyen TH. Topological optimization for designing patient-specific large craniofacial segmental bone replacements. Proc Natl Acad Sci. 2010;107: 13222–13227. 10.1073/pnas.1001208107 20628014 10.1073/pnas.1001208107 PMC2922124

[16] Fraldi M, Esposito L, Perrella G, Cutolo A, Cowin SC. Topological optimization in hip prosthesis design. Biomech Model Mechanobiol. 2010;9: 389–402. 10.1007/s10237-009-0183-0 20037769 10.1007/s10237-009-0183-0

[17] Schweitzer R, Zelzer E, Volk T. Connecting muscles to tendons: tendons and musculoskeletal development in flies and vertebrates. Development. 2010;137: 2807–17. 10.1242/dev.047498 20699295 10.1242/dev.047498 PMC2938915

[18] Edom-Vovard F, Schuler B, Bonnin M-A, Teillet M-A, Duprez D. Fgf4 positively regulates scleraxis and tenascin expression in chick limb tendons. Dev Biol. Academic Press; 2002;247: 351–366. 10.1006/dbio.2002.0707 12086472 10.1006/dbio.2002.0707

[19] Eloy-Trinquet S, Wang H, Edom-Vovard F, Duprez D. Fgf signaling components are associated with muscles and tendons during limb development. Dev Dyn. Wiley‐Liss, Inc.; 2009;238: 1195–1206. 10.1002/dvdy.21946 19384958 10.1002/dvdy.21946

[20] Blitz E, Sharir A, Akiyama H, Zelzer E. Tendon-bone attachment unit is formed modularly by a distinct pool of Scx- and Sox9-positive progenitors. Development. 2013;140: 2680–90. 10.1242/dev.093906 23720048 10.1242/dev.093906

[21] Storm EE, Kingsley DM. GDF5 coordinates bone and joint formation during digit development. Dev Biol. 1999;209: 11–27. 10.1006/dbio.1999.9241 10208739 10.1006/dbio.1999.9241

[22] Kavanagh E, Church VL, Osborne a. C, Lamb KJ, Archer CW, Francis-West PH, et al Differential regulation of GDF-5 and FGF-2/4 by immobilisation in ovo exposes distinct roles in joint formation. Dev Dyn. 2006;235: 826–834. 10.1002/dvdy.20679 16425226 10.1002/dvdy.20679

[23] Schlegel W, Albrecht C, Eckl P, Freudenthaler H, Berger A, Vécsei V, et al Dedifferentiation of human articular chondrocytes is associated with alterations in expression patterns of GDF-5 and its receptors. J Cell Mol Med. 2009;13: 3398–404. 10.1111/j.1582-4934.2009.00953.x 19874419 10.1111/j.1582-4934.2009.00953.x PMC4516495

[24] Giori NJ, Beaupré GS, Carter DR. Cellular shape and pressure may mediate mechanical control of tissue composition in tendons. J Orthop Res. 1993;11: 581–591. 10.1002/jor.1100110413 8340830 10.1002/jor.1100110413

[25] Wilkinson J. Fracture of the patella treated by total excision. A long-term follow-up. J Bone Joint Surg Br. 1977;59: 352–4. Available: http://www.ncbi.nlm.nih.gov/pubmed/893514 893514 10.1302/0301-620X.59B3.893514

[26] Carter DR, Blenman PR, Beaupre GS. Correlations between mechanical-stress history and tissue differentiation in initial fracture-healing. J Orthop Res. 1988;6: 736–748. 10.1002/jor.1100060517 3404331 10.1002/jor.1100060517

[27] Drachman DB, Sokoloff L. The role of movement in embryonic joint development. Dev Biol. 1966;14: 401–420. 10.1016/0012-1606(66)90022-4

[28] Wren T a, Beaupré GS, Carter DR. Mechanobiology of tendon adaptation to compressive loading through fibrocartilaginous metaplasia. J Rehabil Res Dev. 2000;37: 135–143. 10850819

[29] Aslan H, Kimelman-Bleich N, Pelled G, Gazit D. Molecular targets for tendon neoformation. J Clin Invest. 2008;118: 439–444. 10.1172/JCI33944 18246194 10.1172/JCI33944 PMC2214706

[30] Geris L, Vander Sloten J, Van Oosterwyck H. In silico biology of bone modelling and remodelling: regeneration. Philos Trans R Soc A Math Phys Eng Sci. 2009;367: 2031–2053. 10.1098/rsta.2008.0293 19380324 10.1098/rsta.2008.0293

[31] Wren T a Beaupré GS, Carter DR. Mechanobiology of tendon adaptation to compressive loading through fibrocartilaginous metaplasia. J Rehabil Res Dev. 2000;37: 135–143. 10850819

[32] Piszcatowski S. Material aspects of growth plate modelling using Carter’s and Stokes’s approaches. Acta Bioeng Biomech. 2014;13: 3–14. 22097913

[33] Guevara JM, Moncayo MA, Vaca-González JJ, Gutirrez ML, Barrera LA, Garzón-Alvarado DA. Growth plate stress distribution implications during bone development: A simple framework computational approach. Comput Methods Programs Biomed. Elsevier Ireland Ltd; 2015;118: 59–68. 10.1016/j.cmpb.2014.10.007 25453383 10.1016/j.cmpb.2014.10.007

[34] MacAusland WR. Total excision of the patella for fracture. Am J Surg. 1946;72: 510–516. 10.1016/0002-9610(46)90385-6 20997781 10.1016/0002-9610(46)90385-6

[35] Bruce J, Walmsley R. EXCISION OF THE PATELLA: Some Experimental and Anatomical Observations. JBJS. 1942;24. Available: https://journals.lww.com/jbjsjournal/Fulltext/1942/24020/EXCISION_OF_THE_PATELLA__Some_Experimental_and.8.aspx

[36] Sigmund O. A 99 line topology optimization code written in matlab. Struct Multidiscip Optim. 2001;21: 120–127. 10.1007/s001580050176

[37] Sutradhar A, Park J, Carrau D, Nguyen TH, Miller MJ, Paulino GH. Designing patient-specific 3D printed craniofacial implants using a novel topology optimization method. Med Biol Eng Comput. Springer Berlin Heidelberg; 2016;54: 1123–1135. 10.1007/s11517-015-1418-0 26660897 10.1007/s11517-015-1418-0

