# Supplementary material for: Computational model for the patella onset
Source: PLoS One. 2018 Dec 11;13(12):e0207770. doi: 10.1371/journal.pone.0207770 (PMC6289436; doi:10.1371/journal.pone.0207770)
Supplement: S1 Table — (DOCX) [file pone.0207770.s001.docx]

| Variable | Value | | | |  |
| --- | --- | --- | --- | --- | --- |
|  | **Mesenchymal** | **Cartilage** | **Tendon** | **Interzone** | **theory** |
| $\boldsymbol{\sigma}_{\mathbf{hyd}}^{\mathbf{cart}}$ | -0.013 | | | | II |
| $\boldsymbol{D}_{\boldsymbol{TGF-\beta}}$ | 1.0 | 1.0/10 | 1.0/10 | 1.0/10 | I |
| $\boldsymbol{D}_{\boldsymbol{BMP}}$ | 0.05 | 0.05/10 | 0.05/10 | 0.05/10 | I |
| $\boldsymbol{\mu}\left( \boldsymbol{s}_{\boldsymbol{at}}\left( \boldsymbol{x,t} \right) \right)$ | $0.1+0.1*S_{TGF-\beta}$ | $0.1*S_{TGF-\beta}$ | 0.0001 | 0.0001 | I |
| $\boldsymbol{\chi}\left( \boldsymbol{s}_{\boldsymbol{at}}\left( \boldsymbol{x,t} \right) \right)$ | 0.5 | 0.0005 | 0.0005 | 0.0005 | I |
| $\boldsymbol{D}_{\boldsymbol{FGF}}$ | 7.0/1000 | 7.0/1000 | 7.0 | 7.0/1000 | I |
| $\boldsymbol{D}_{\boldsymbol{GDF-5}}$ | 0.05 | 0.05/1000 | 0.05/10 | 0.05/10 | I |
| $\boldsymbol{b}_{\boldsymbol{c}}^{\boldsymbol{Th}}$ | 0.65 | | | | I |
| $\boldsymbol{S}_{\boldsymbol{BMP}}^{\boldsymbol{Th}}$ | 0.03 | | | | I |
| $\boldsymbol{S}_{\boldsymbol{FGF}}^{\boldsymbol{Th}}$ | 0.1 | | | | I |
| $\boldsymbol{S}_{\boldsymbol{GDF-5}}^{\boldsymbol{Th}}$ | 0.038 | | | | I |
